# Phyllotaxis transition over the lifespan of a palm tree using Magnetic Resonance Imaging (MRI) and Terrestrial Laser Scanning (TLS): the case of *Jubaea chilensis*

**DOI:** 10.1186/s13007-022-00920-z

**Published:** 2022-06-25

**Authors:** Eduardo Guzmán, M. Paulina Fernández, José-Antonio Alcalde, Samuel Contreras, Pasi Raumonen, Lorenzo Picco, Cristián Montalba, Cristián Tejos

**Affiliations:** 1 Master Program in Natural Resources, Facultad de Agronomía e Ingeniería Forestal, Pontificia Universidad Católica de Chile, Santiago, Chile; 2 Facultad de Agronomía e Ingeniería Forestal, Pontificia Universidad Católica de Chile, Santiago, Chile; 3 Centro Nacional de Excelencia para la Industria de la Madera (CENAMAD), Pontificia Universidad Católica de Chile, Santiago, Chile; 4 Centro UC de Innovación en Madera, Pontificia Universidad Católica de Chile, Santiago, Chile; 5 Computing Sciences, Tampere University, Tampere, Finland; 6 Department of Land, Environment, Agriculture and Forestry, Universitá degli Studi di Padova, Padua, Italy; 7 Biomedical Imaging Center, Pontificia Universidad Católica de Chile, Macul, Santiago, Chile; 8 Radiology Department, School of Medicine, Pontificia Universidad Católica de Chile, Santiago Centro, Santiago, Chile; 9 Department of Electrical Engineering, Pontificia Universidad Católica de Chile, Macul, Santiago, Chile; 10 Millennium Institute for Intelligent Healthcare Engineering (iHEALTH), Santiago, Chile

**Keywords:** Arecaceae, Fibonacci sequence, Magnetic Resonance Imaging, Phyllotaxis, Terrestrial Laser Scanning, *Jubaea chilensis*, Divergence angle, Parastichy

## Abstract

**Background:**

*Jubaea chilensis* (Molina) Baillon, is a uniquely large palm species endemic to Chile. It is under threatened status despite its use as an ornamental species throughout the world. This research seeks to identify the phyllotaxis of the species based on an original combination of non-destructive data acquisition technologies, namely Magnetic Resonance Imaging (MRI) in saplings and young individuals and Terrestrial Laser Scanning (TLS) in standing specimens, and a novel analysis methodology.

**Results:**

Two phyllotaxis parameters, parastichy pairs and divergence angle, were determined by analyzing specimens at different developmental stages. Spiral phyllotaxis patterns of *J. chilensis* progressed in complexity from parastichy pairs (3,2) and (3,5) in juvenile specimens and (5,3), (8,5) and (8,13) for adult specimens. Divergence angle was invariable and averaged 136.9°, close to the golden angle. Phyllotactic pattern changes associated with establishment phase, the adult vegetative and the adult reproductive phases were observed. Both technologies, MRI and TLS proved to be adequate for the proposed analysis.

**Conclusions:**

Understanding phyllotactic transitions may assist identification of developmental stages of wild *J. chilensis* specimens*.* The proposed methodology may also be useful for the study of other palm species.

## Introduction

Morphological patterns in plants, such as phyllotaxis, are the result of the genetics and physiology of the species and their adaptation to the environment [1, 2]. The study of these patterns allows not only the classification of the species, but also the understanding of trade-offs among different functional, biomechanical and morphological aspects. Furthermore, phyllotaxis is an important variable in development and growth models of species under different environments [3–5].

**Fig. 1:**
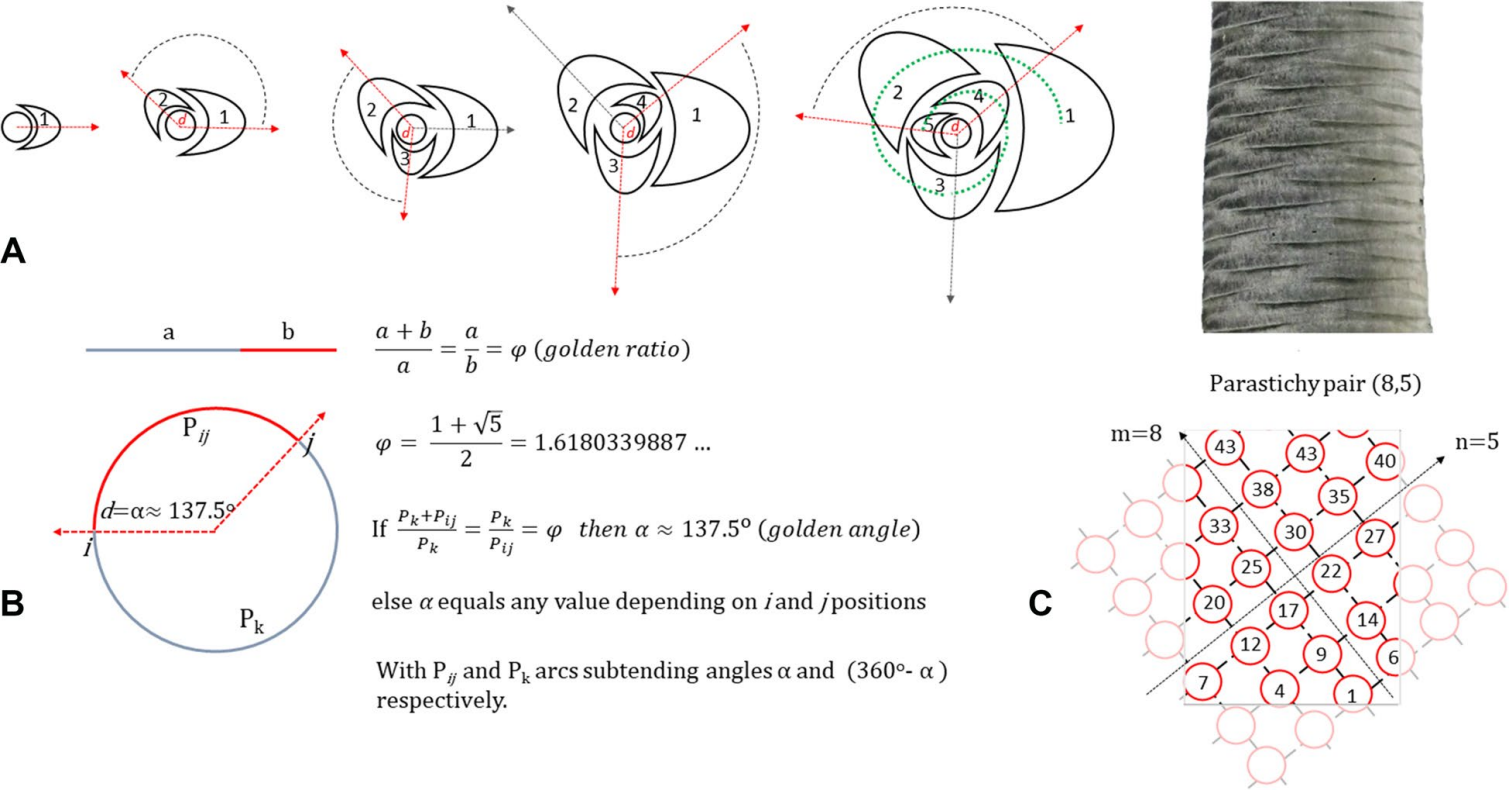
**A** Leaf sequence at the apex, following a certain divergence angle *d* (genetic spiral in dotted line; **B** relationship between the golden ratio and the golden angle, where P_k_ and P_ij_ are respectively the long and short arclengths that give rise to the golden ratio; **C** the left and right-handed spiral formed in a stem lattice resulting from petiole scars and its generated (8,5) parastichy pairs

Two parameters have been widely used for an objective study of phyllotaxis [6–10]: divergence angle and parastichy. The divergence angle, *d*, is the enclosed angle between two successive lateral organs (buds, shoots, leaves, labelled *ij* in Fig. 1B) arising from the apical meristem of an axis. In plants displaying spiral phyllotaxis, *d* tends to be approximatively α ≈ 137.5° (Fig. 1B), which is called the golden angle. This name comes from the corresponding golden ratio, extensively studied during the Italian Renaissance [11–16]. Parastichy refers to a secondary spiral that connects a sequence of leaves. In plants with spiral or helix phyllotaxis, if primordia are numbered in consecutive order from the apical meristem, the visual connection of those consecutive primordia is called the “genetic helix”, “ontogenic spiral”, or “genetic spiral” [14, 17] (Fig. 1A, at right, green line). Nevertheless, when a plant presents a spiral phyllotaxis, other spiral sequences might be observed. Thus, a parastichy corresponds to a visual secondary (Fig. 1C)—but not necessarily a genetic-spiral.

Visual spirals go in both, clockwise and counterclockwise directions and intersect at an angle of approximately 90° (Fig. 1C). The number of left (*m*) and right (*n*) spiral is called a *parastichy pair* (*m,n*) [15, 17]. These *m* and *n* are usually consecutive numbers of the well-known mathematical Fibonacci sequence [14, 18–20]. When observed in a lattice, as it occurs when observing a stem from the side, the *m* and *n* values are the difference between contiguous numbers from the genetic spiral, generated by the secondary left-handed spiral (*m*) and the right-handed spiral (*n*). Thus, the left-handed spiral will connect every *m*th leaf, while the right-handed spiral will connect every *n*th leaf. In Fig. 1C, for example, the left-handed spiral connects every 8th leaf, while the right-handed spiral connects every 5th leaf. Therefore, the parastichy pair is (8,5). For more details, we refer to Mitchinson [14].

Morphological patterns can change during the lifespan of an individual, depending on the initial morphological structure and growth rate; in particular, the transition from juvenile to adult phase can be followed by changes in phyllotaxis [16, 19–21]. In this case, quantification of the divergence angle and the parastichy can be means to determine breakpoints in the phase changes of individuals, or to determine transitional stages during development and maturation of a plant.

Palm trees are among the most intriguing plants. Although they are a monocot species, they can reach as high as 30 m, with diameters > 1 m, based entirely on primary growth [22, 23]. The spiral phyllotaxis of their leaves usually follows the first part of the Fibonacci sequence (1, 1, 2, 3, 5, 8…) with the resulting (*m, n*) pairs: (1,2), (2,3), (3,5), (5,8), … [24, 25]. These arrangements are visible on the palms in three ways: scars from fallen leaves, the crown itself and in the apex [25]. The divergence angle tends to be regular, but there may be some variability within the same individual [10].

Among palm trees, we decided to study *Jubaea chilensis* (Molina) Baill. (Arecales: Arecaceae), commonly known as the Chilean palm, an endemic palm of Central Chile, because the species is one of the most massive palms in the world (it is among the largest 4% of palm trees), with heights up to 33 m and stem diameters of 2 m in wild populations. Additionally, it has the southernmost distribution of all palm species in the Americas down to approximately 35° S latitude. *J. chilensis* inhabits a Mediterranean climate enduring very hot summer events as well as winter snow. Therefore, the species has been used as an ornamental plant in America (such as USA, Perú, Bolivia, Argentina), Europe (such as Italy, France, Portugal, Germany, Ireland, among others), Russia, New Zealand and Australia [26].

During the first 20 years, the stem of the Chilean palm grows mainly in diameter, after which it increases in height. Its perennial leaves are pinnate in the adult phase and grouped in the upper stem forming a dense crown, with a sequential turnover of 8 to 12 leaves per year in adult individuals. Although *J. chilensis* is a unique species, and is highly considered as an ornamental plant worldwide, there is a lack of knowledge about its biology and morphology. Studies have been focused mainly on germination treatments, nursery techniques and establishment protocols [26, 27]. Thus, efforts to understand its phyllotaxis and phase change evolution itself is a contribution to its knowledge and a challenge we decided to accomplish using Magnetic Resonance Imaging (MRI) and Terrestrial Laser Scanning (TLS). We postulate these two non-destructive approaches as complementary techniques to acquire respectively internal and external information which might be used in phyllotaxis studies.

### Magnetic Resonance Imaging (MRI) and Terrestrial Laser Scanning (TLS) technologies applied to the measurement of plant structures

Non-destructive testing and remote data acquisition of biological structures are quite important when manipulation of the specimen may change the characteristics of interest, when the sample is in a position that is difficult to access or when the specimen is alive. In the present research we tested two technologies to help overcome these difficulties in juvenile and adult palm specimens.

Magnetic Resonance Imaging (MRI) can be used to survey plant specimens [28, 29]. An MRI scanner consists of a superconducting magnet, which produces a strong homogeneous magnetic field of up to 21 Tesla (T) that polarizes the single proton Hydrogen (^1^H) (or other elements, depending on the analysis). Radio-Frequency (RF) and magnetic gradient coils are turned on and off, exciting and relaxing the proton spins. The readout of the recovery to the equilibrium orientation of the spins allows 3D image reconstruction of the scanned object, based on different properties such as ^1^H proton density (ρ), longitudinal relaxation time constant (T_1_) and transverse relaxation time constant (T_2_). A 3D voxel arrangement, normally in grey-scale, is produced and can be explored in any direction. The desired measurements are done on one or more intersecting planes in a 3D space. Given the significant presence of water, MRI technology has been used for physiological and morphological studies of plants, including tissues and organs such as wood, roots, leaves, and fruits [28–35] among others.

Terrestrial Laser Scanning (TLS) is a time-of-flight (first return) scanner. It consists of a ground-based LiDAR (light detection and ranging) technology that scans, the surface of objects. It produces a dense cloud of 3D points by measuring the time taken for a laser pulse to reach different locations on the target surface and return to the sensor (laser head), assuming a known speed of light [36]. TLS provides measurements with a precision of 6 mm for position and of 4 mm for distance at a 100 m distance, and a speed of up to 50,000 points per second of the scanned surface, allowing rapid and automatic acquisition of detailed data of trees and forest [37]. Particularly, it allows the acquisition of data from high locations on standing trees, where it is difficult to reach or extremely time-consuming. Most studies of the crown architecture and structure at different positions of the trunk used felled trees [38, 39]. This is reasonable when the species is rather common and little harm is done when felling some individuals, for instance in productive plantations. However, the option of felling is neither possible nor desirable when working with endangered species with small extant populations or with individuals of remarkable value, such as old individuals, or in an urban context. TLS has been increasingly used for reconstructing tree structures [40–43], inventorying a forest [37, 44, 45] and studying urban trees [46–48]. TLS technology greatly improves the possibility to enlarge quantitative analyses on standing trees and forest structures, moving ahead from mere qualitative reconstructions. TLS appears as the most accurate among various image or point cloud techniques [20]. Some of the most relevant limitations of this technology are the lack of mobility, the lack of automatic tree species classification, obtaining sample points only from the surface of the analysed specimen, which may be affected by occlusion from other objects that obstruct direct view.

This research aimed at identifying the two phyllotactic parameters, divergence angle and parastichy values, of *J. chilensis* specimens at different developmental stages and to analyze any phyllotactic transition during the lifespan of the species. The phyllotaxis and its transitions can be related to different maturity stages of plants and different functionalities. The state of this palm and other endangered species prompted us to look for these innovative and non-destructive sampling techniques based on MRI and TLS, developing some particular methodologies for the data acquisition and analysis.

## Materials and methods

### Determination of the phyllotactic parameters of young *J. chilensis* plants

Four young *J. chilensis* plants (Jch1–Jch4) were used to identify the phyllotactic parameters of divergence angle and parastichies. The plants were obtained from the “Oasis de la Campana” Nursery, north of Santiago (32° 54′ 35″ S; 71° 04′ 58″ W), from seeds originally collected from a natural population of palm trees close to the nursery and the Hijuelas town, Region of Valparaíso, Chile. This population is limiting with the largest and most important remaining populations of *J. chilensis*, protected in the Ocoa National Park [49], under a climate regime of annual mean rainfall of 338 mm year^−1^, annual mean temperature of 14.5 °C, with an average minimum temperature of the coldest month of 4.3 °C in July, and average maximum temperature of the warmest month of 26.6 °C in January (Runge DGA weather station [50]).

These four individuals were preliminarily characterized (Table 1). The plants were cultivated in polyethylene bags of different sizes, depending on the age and dimension of the individuals, using a standard, well-drained growing media, with regular irrigation and nutritional supplementation. Although the oldest plant of this group was a 13-year-old specimen, the stem and total height were rather low; this is typical for the species, as plants remain relatively small while increasing in diameter during the first years before growing in height [51].

**Table 1:** Characteristics of four *Jubaea chilensis* specimens (Jch1–Jch4) analyzed by Magnetic Resonance Imaging (MRI) and one adult specimen (Jch5) analyzed by Terrestrial Laser Scanning (TLS)

Specimens	Age (years)	Basal diameter (cm)	Stem height (cm)	Total height (cm)	Number of leaves
Jch1	4	1.6	4	46	7
Jch2	5	1.9	5	26	7
Jch3	7	4.5	5.5	44	6
Jch4	13	5.1	12	62	10
Jch5	160 (estimated)	103	1380	1620	> 58

MRI allows the detection of internal structures and tissues in a 3D framework, without destroying the arrangement of the structures, their turgor, size or function, due to the minimal manipulation of the individual. In the context of the present study, MRI allows the analysis of the internal structure at the meristem such as the arrangement of leaf primordia, some of which are not yet visible to the naked eye. Tomographic images as those obtained from MRI simplify measurement procedures and offer greater accuracy than cutting, extracting, and measuring structures by traditional anatomical approaches. The juvenile palm trees (Jch1 to Jch4) were scanned using a 1.5 Tesla whole-body MRI scanner (Phillips Achieva, Best, The Netherlands) with a cardiac surface 5-element phase array coil. Palm trees were introduced lying down to the scanner. A multi-slice (i.e., a series of 2D slices) turbo spin echo sequence was used to acquire the palm tree images, with the following acquisition parameters for all scans: axial slice orientation, repetition time (TR) 2000 ms, echo time 30 ms, turbo factor 6, in-plane acquisition resolution 0.7 × 0.7 mm^2^, in-plane reconstructed resolution 0.35 × 0.35 mm^2^, acquired and reconstructed slice thickness 3 mm, gap between consecutive slices 0.5 mm, number of sampled averages 8. Those acquisition parameters that are specific for each palm tree are summarized in Table 2.

**Table 2:** MRI acquisition parameters

MRI parameters	Jch1	Jch2	Jch3	Jch4
In-plane field of view (mm)	120 × 120	70 × 70	70 × 70	50 × 50
In-plane matrix (pixels)	170 × 170	100 × 100	100 × 100	72 × 72
Slices	27	18	18	18
Bandwidth (Hz)	183.1	188.6	185.4	191.6
Acquisition time	7 min 32 s	9 min 8 s	8 min 36 s	6 min 28 s

**Fig. 2:**
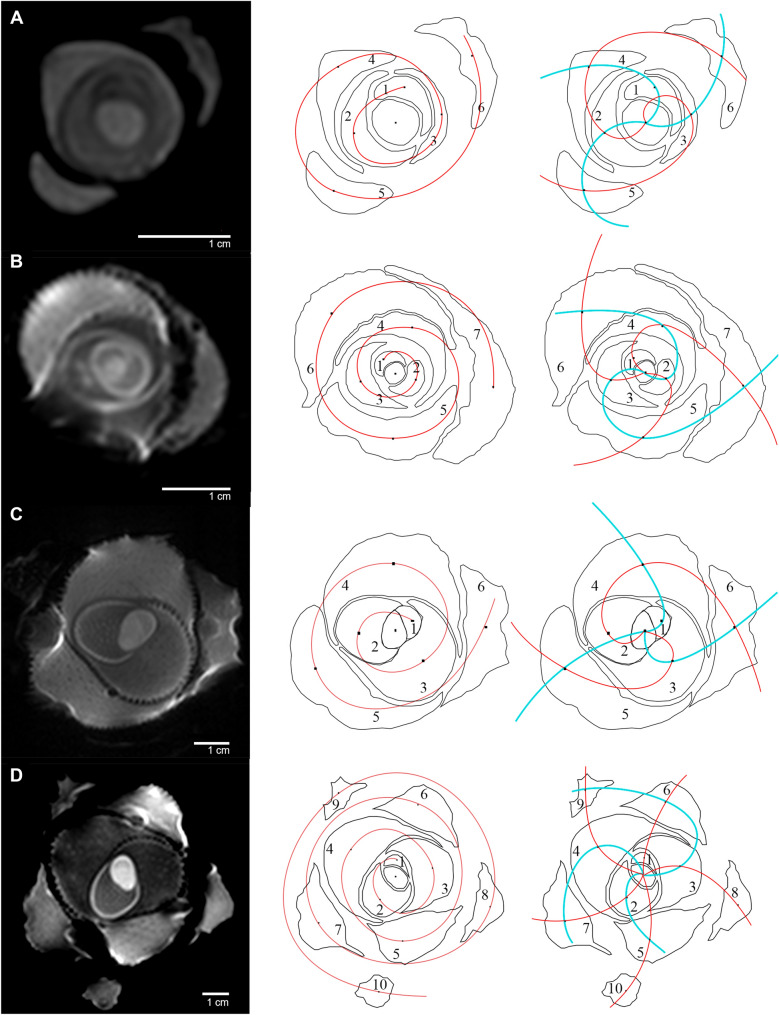
MRI axial images (left), generative spiral (center) and parastichies (right) of juvenile *J. chilensis* specimens. **A** A four-year-old specimen (Jch1) with a (3,2) parastichy pair and a counterclockwise direction genetic spiral; **B** a five-year-old specimen (Jch2) with a (2,3) parastichy pair and a clockwise direction genetic spiral; **C** a 7-year-old specimen (Jch3) with a (3,2) parastichy pair and a counterclockwise direction genetic spiral; and **D** a 13-year-old specimen (Jch4) with a (3,5) parastichy pair and a counterclockwise direction genetic spiral

The parastichies were analyzed from the axial images of each palm. In the most suitable images, the identified structures were delimited and schematized, and a central point of each foliar primordium was manually fixed in the thickest region of each foliar structure (Fig. 2, dots on the second and third column schemes). The structures were then numbered from the interior to the exterior, following the genetic spiral, the central structures being younger than the external ones (Fig. 2, second column, showing the genetic spiral in a solid red line).

Conspicuous parastichy pairs were identified connecting the central points of the neighboring structures in both directions (clockwise and counterclockwise) in accordance with Jean’s definition [10, 18] (Fig. 2, third column, red and cyan lines show clockwise and counterclockwise spirals respectively). The divergence angle (*d*) was determined as the angle formed between two consecutive structures, measured from the lines projected from the center of the apex to the structure’s central point. These measurements were carried out from the youngest to the oldest foliar primordium.

### Determination of phyllotactic parameters in adult *J. chilensis* plants

An adult (reproductive) specimen of *J. chilensis* (Jch5) in the historical urban park of Quinta Normal in Santiago (33° 26′ 26″ S, 70° 40′ 58″ W) was assessed for the phyllotactic parameters. The specimen was 13.8 m high to the apex (16.2 m to the highest leaf) and grows in a Mediterranean environment, with 310 mm year^−1^ of rainfall, annual mean temperature of 14.6 °C, with an average minimum temperature of the coldest month of 3.8 °C in July, and an average maximum temperature of the warmest month of 29.9 °C in January (Quinta Normal DGA weather station [50]). The individual was under adequate watering (Fig. 4A).

The stem surface of the palm was scanned using a Leica ScanStation2 TLS (Leica Geosystems AG, Heerbrugg, Switzerland) provided by the Department of Land, Environment, Agricultural, and Forestry of the University of Padova, Italy. Several scans from different positions generated a dense and well-distributed cloud of points over the trunk surface.

**Fig. 3:**
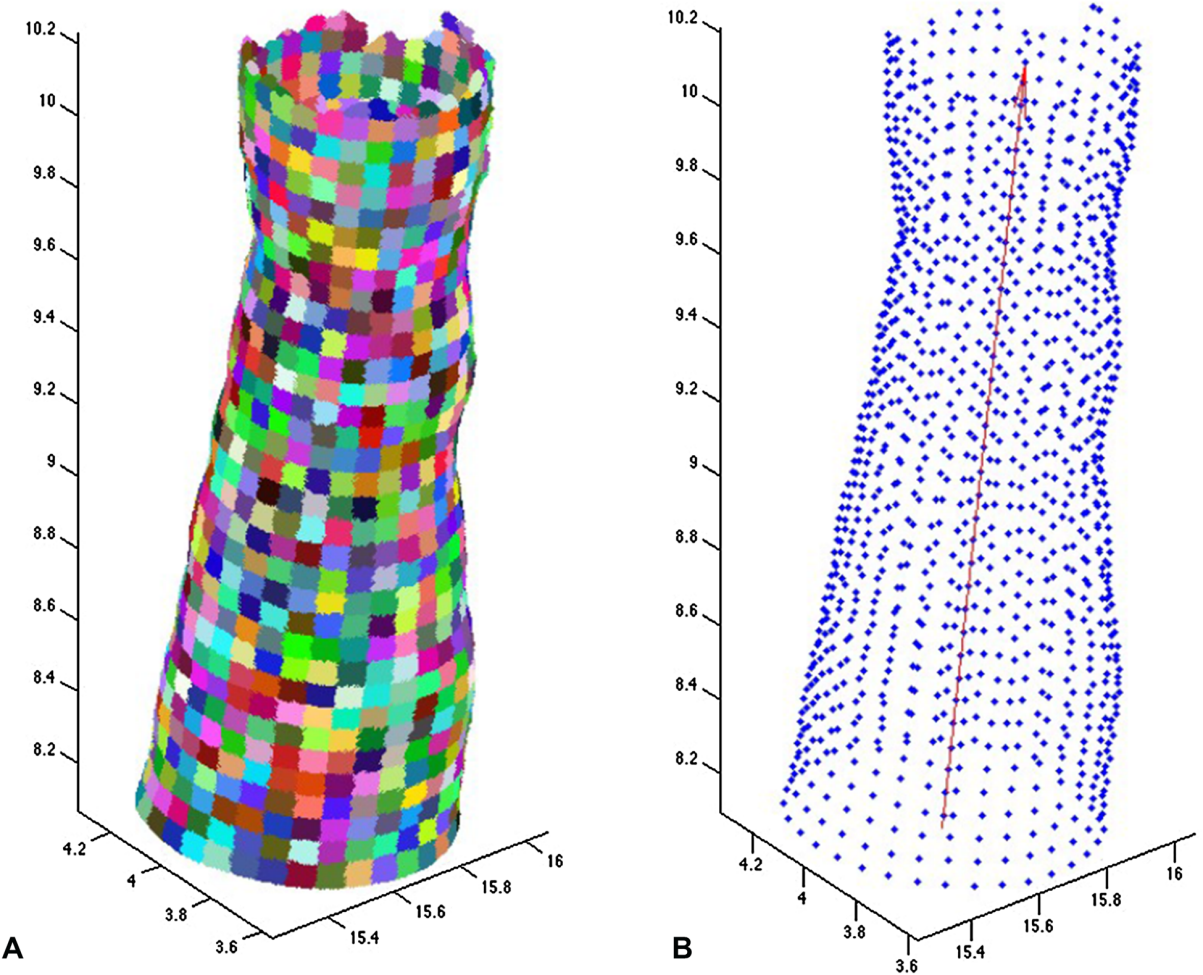
Smooth surface model of the stem produced from a TLS cloud of points. **A** The model consists of small patches; **B** to simplify the quantitative analysis, an approximate axis as a straight line close to the center of the stem, going from the bottom to the top was defined (red arrow), and each patch is represented by a single blue point. For illustrative purposes, we have increased the size of the patches of our surface model

**Fig. 4:**
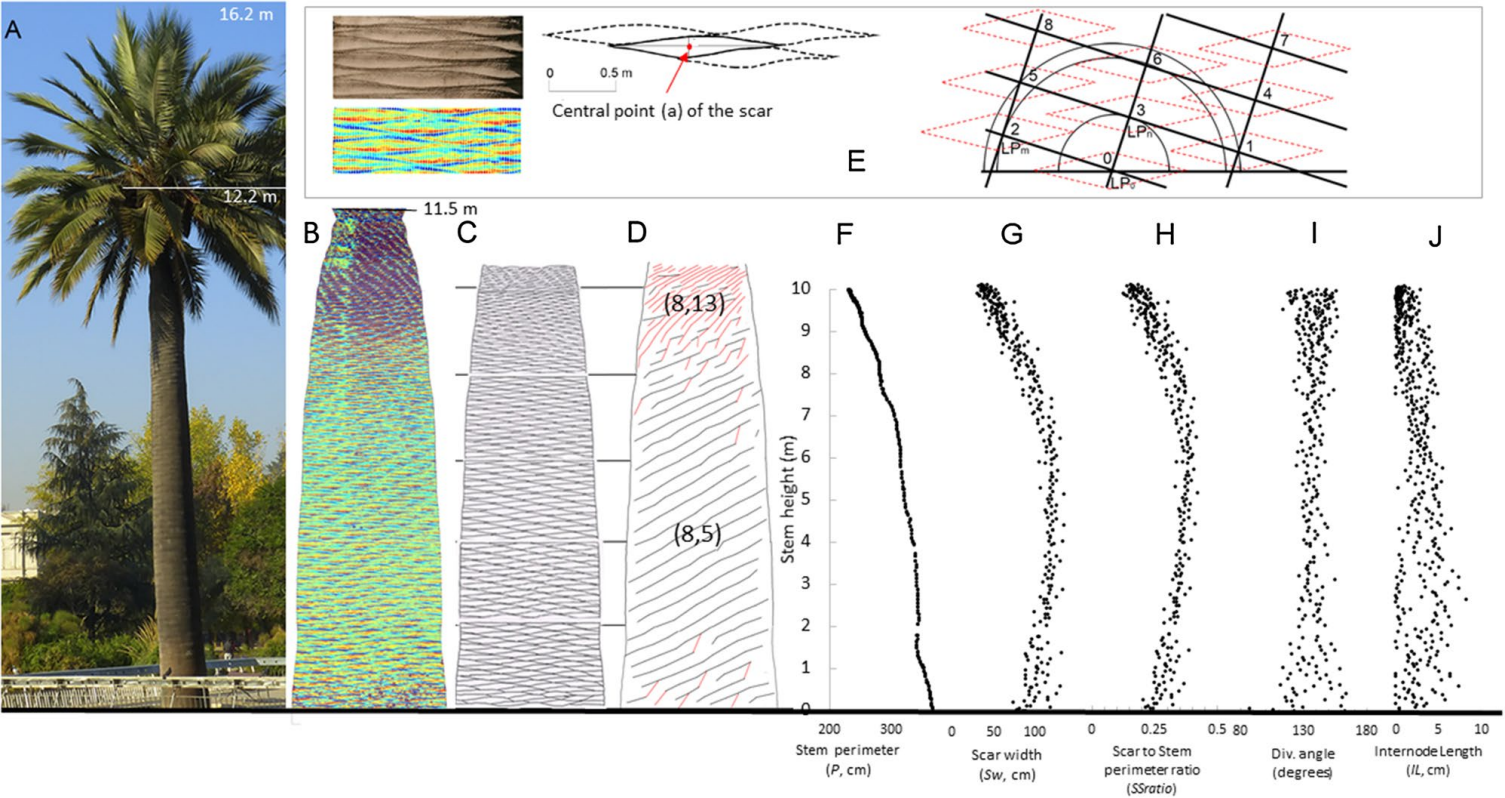
**A** Adult *J. chilensis* specimen (Jch5); **B** 2D projection of the TLS data and after applying the surface model algorithm; **C** delimited scars of the specimen; **D** representation of the parastichies: black lines represent (8,5) and red lines represent (8,13) (only right-handed parastichies are shown); **E** magnified view of the scars on the stem of *J. chilensis*; section of the planar projection of the stem showing the limits of the scars; final delimitation of leaf scars in the adult specimen of *J. chilensis*, with the definition of the central point of each scar, *a*, as the intersection between its largest vertical and horizontal dimension; numbering system of scars and definition of the parastichy; **F** stem perimeter (*P*, cm) profile; **G** scar width (*Sw*, cm) along the stem; **H** Scar to Stem perimeter ratio along the stem (*SSratio*); **I** divergence angle along the stem; **J** internode length (*IL*, cm) along the stem

Petiole abscission scars on the palm stem were segmented by analyzing the radius value of the points on the stem surface. To analyze these radii variations, we created a smooth 3D surface model as follows: the point cloud was partitioned into small patches assuming a cylindrical coordinate system (Fig. 3A). To create those patches, we first defined an approximate axis as a straight line close to the center of the stem, going from bottom to top (red arrow in Fig. 3B). Patches were defined considering azimuth (i.e. the angle in cylindrical coordinates) and axial distance. In the azimuth direction, we considered 288 divisions, each of them at 1.25°. Along the axial direction, we considered divisions in steps of 2.5 mm. Each subset of measured points belonging to the same patch was replaced with its average value, shown as blue points in Fig. 3B, to produce the smooth 3D surface model. The surface model formed a topologically regular grid of points where each point has the same kind of neighborhood. The surface model was smoothed by averaging over a 5 × 5 point moving window. The 3D surface model was projected onto a 2D plane to produce images for further analysis, where the angular and axial coordinates were the x- and y-coordinates on the plane, respectively (Fig. 4B). The coordinates were also adjusted in the projection to maintain the true scale (perimeter length at any height) of the stem surface. To facilitate the analysis, this 2D plane was divided into six sections along the axial direction (Fig. 4B, C).

In the planar projection, the leaf scars (Fig. 4B, E) were identified as rhomboids, bounded by an upper and a lower line (Fig. 4E). The central point (a) of the scar, expressed in Euclidian coordinates (*x*,*y*) of the planar projection, was manually defined as the intersection between the longest horizontal and vertical rhomboid axes (Fig. 4E). The order of appearance of each scar was defined based on the *y*-coordinate of point (a), with the lowest labelled as number 1. Using the central point of the scars, the species parastichies were identified following Jean’s definition [18], that is: (1) a parastichy pair is considered visible if there is a lattice point at each intersection of the parastichies from the left-handed and right-handed spirals; (2) a pair (*m,n*) is considered conspicuous if *LP*_*m*_ (Lattice Point *m*) and *LP*_*n*_ are the closest lattice points to any *LP*_*0*_; and (3) a lattice is said to be of type (*m,n*), if (*m,n*) is a conspicuously visible pair (Fig. 4E). This procedure was robust to identify the scars up to 10 m height (Fig. 4C, D). Above that height, scars get thinner and their boundaries become diffuse. The correspondence between the TLS borders of the scars and the actual limits of the scar was verified by comparing data from some stem sections with in situ photographs and taking direct measurements performed on the scars.

The divergence angle (*d*) between two consecutive scars was calculated as

1 $$d = \left( {{{P_{ij} } \mathord{\left/ {\vphantom {{P_{ij} } P}} \right. \kern-\nulldelimiterspace} P}} \right)*{36}0,$$

where *P* is the total stem perimeter at the corresponding height, and *P*_*ij*_ is the arc length of the segment between the two consecutive scars *i* and *j*. The latter is equivalent to the horizontal distance in the planar projection of the central point (a) of two consecutive scars (Fig. 1B).

To study the transition of the parastichy pair of an adult specimen in relation to the stem narrowing zone, we evaluated the Scar to Stem ratio *SSratio*_*ih*_ (Eq. 2). This is the ration between the scar width *i* at height *h* (*Sw*_*ih*_) (Fig. 4E, G) and the corresponding stem perimeter (*P*_*h*_) (Fig. 4F). This was performed as proposed by Atela [5] and Bryntsev [52], assuming that changes in parastichy depend not only on changes in divergence angle, but also on changes in organs and stem perimeters.

2 $$SSratio_{ih} = \left( {{{Sw_{ih} } \mathord{\left/ {\vphantom {{Sw_{ih} } {P_{h} }}} \right. \kern-\nulldelimiterspace} {P_{h} }}} \right).$$

Additionally, we computed the internode length (*IL*_*ij*_, cm), as the vertical distance between two consecutive scars *i* and *j*, this is,

3 $$IL_{ij} = H_{j} {-}H_{i} ,$$

where *H*_*i*_ and *H*_*j*_ are the height of the central point (a) of consecutive scars *i* and *j,* respectively.

Divergence angle (*d*) using data from all palms were grouped by parastichy classes, [class (3,2) including (2,3) and (3,2) parastichy pairs; class (3,5) including (3,5) and (5,3) parastichy pairs; class (8,5); class (8,13)]. To test whether the divergence angle exhibits differences related to the kind of parastichy pair, we performed a non-parametric Kruskal–Wallis test by ranks of divergence angles between classes of parastichy pairs.

In the adult specimen, divergence angle (*d*), Scar width (*Sw*), stem perimeter (*P*), Scar to Stem ratio (*SSratio*) and internode length (*IL*) were also grouped into the two main classes of parastichy pairs [(8,5) and (8,13)] and contrasted in the same way. Additionally, in the adult specimen, the divergence angle trend was evaluated using a linear regression of the form,

4 $$d_{i} = a \cdot H_{i} + b,$$

where *d*_*i*_ is divergence angle of the scar *i* and *H*_*i*_ is the height of the central point (a) of scar *i*. Data analysis was performed using the R Package [53].

## Results

### Determination of phyllotaxis parameters: divergence angle and parastichies

MRI images of the young *J. chilensis* individuals showed the distribution of the expanding and mature leaves (Fig. 2). The generative spirals of three of these specimens (Fig. 2, specimens A, C and D) ran counterclockwise and one ran clockwise (Fig. 2B). Whereas for the fifth specimen (Jch5), the planar projection of the TLS data allowed individualizing and measuring each scar on the stem perimeter and calculating the divergence angle and the parastichy series followed by the visual spirals.

Table 3 summarize the divergence angles and parastichy pairs that define the phyllotaxis of the studied specimens. In the juvenile specimens (Jch1 to Jch4), the divergence angles ranged between 92.1° and 176.3°, with an average of 139.5°. Specimen Jch5 had an average divergence angle of 136.7°, with a range between 105.8° and 163.5°. Divergence angles along the stem of Jch5 are shown in Fig. 4I.

**Table 3:** The average divergence angle and phyllotaxis of five *J. chilensis* specimens

Specimen	Growth stage (years)	Parastichy pairs	Height range (m)	Average divergence angle (SD)	Min. divergence angle	Max. divergence angle	Coefficient of variation (CV%)	N
Jch1	4	(3,2)		136.8° (36.7)	92.1°	176.3°	26.8	5
Jch2	5	(2,3)		143.2° (14.1)	123.1°	162.6°	9.8	6
Jch3	7	(3,2)		138.7° (17.8)	113.6°	156.9°	12.8	5
Jch4	13	(3,5)		138.9° (8.6)	126.8°	156.3°	6.2	9
Overall mean of juvenile specimens				139.5° (18.7)	92.1°	176.3°	13.4	25
Jch5		(5,3)	0–0.24	128.8° (21.2)	105.8°	162.2°	16.4	8
Jch5		(8,5)	0.24–8.97	137.0° (8.4)	111.3°	163.5°	6.1	321
Jch5		(8,13)	8.97–10.18	136.5° (11.0)	113.0°	157.3°	8.0	133
Jch5	Adult	Total	0–10.18	136.7° (9.6)	105.8°	163.5°	6.9	462
Overall mean value				136.9° (10.2)	92.1°	176.3°	7.5	487

*SD* Standard deviation

The phyllotaxis of the first three specimens (Jch1 to Jch3) was defined by parastichy pairs (3,2) or (2,3) (Fig. 2A–C), whereas that of the fourth (Jch4) was (3,5) (Fig. 2D). All these pairs are numbers from the Fibonacci series. The leaves followed spiral phyllotactic patterns: a genetic spiral (red lines, second column of Fig. 2) and two visual spirals (red and cyan lines, third column of Fig. 2).

The parastichy pairs of the adult Chilean palm specimen (Jch5) were identified in the planar projection of the TLS points. Three parastichy pairs were observed: (5,3), (8,5) and (8,13), all corresponding to consecutive numbers of the Fibonacci series. In Fig. 4D only the right-handed parastichies are shown: (8,5) as black lines and (8,13) as red lines. The phyllotactic pattern (8,5) dominates up to 8.97 m in height, followed by a phyllotactic pattern (8,13) (Table 3). Since the (5,3) pattern only occurs on a short basal section of 8 scars, our analysis was focused on regions with (8,5) and (8,13) parastichy pairs.

### Characterization of phyllotaxis parameters in the adult *J. chilensis* palm

The studied stem parameters of the adult *J. chilensis* specimen (*Sw*, *P*, *SSratio*, *IL*, *d*) are presented in Table 4, either for the overall range or separated in the two main phyllotaxis sections.

**Table 4:** Scar width (*Sw*), stem perimeter (*P*), scar to stem ratio (*SSratio*), internode length (*IL*) and divergence angle (*d*) of the adult *J. chilensis* (Jch5)

Variable		Average (SD)	Min	Max	CV (%)	N	Chi-square	p-value
*Sw* (cm)	Overall	88.02 (29.8)	17.5	135.7	33.90	487	272.7	2.2e−16
	(8,5) section	104.36 (17.4)	55.4	135.7	16.67	321		
	(8,13) section	48.47 (11.97)	17.5	77.7	24.69	133		
*P* (cm)	Overall	294.7 (42.18)	226.1	363.7	14.31	487	281.5	2.2e−16
	(8,5) section	316.5 (29.05)	258.2	363.7	9.18	321		
	(8,13) section	241.9 (9.74)	226.1	258.2	4.02	133		
*SSratio*	Overall	0.29 (0.074)	0.07	0.42	25.59	487	254.73	2.2e−16
	(8,5) section	0.32 (0.074)	0.20	0.42	13.93	321		
	(8,13) section	0.19 (0.040)	0.07	0.30	21.68	133		
*IL* (cm)	Overall	2.2 (1.73)	0.01	8.22	78.49	487	113.04	2.2e−16
	(8,5) section	2.74 (1.73)	0.01	8.22	63.24	321		
	(8,13) section	0.91 (0.78)	0.01	3.87	85.89	133		
*d* (°)	Overall	136.9 (10.2)	92.1	176.3	7.5	487	0.078952	0.7787
	(8,5) section	137.0 (8.4)	111.0	163.5	6.1	321		
	(8,13) section	136.5 (11)	113.0	157.3	8.0	133		

Figure 4F–J also show these variables, alongside with a photograph (Fig. 4A), the TLS data (Fig. 4B) and the stem scheme showing the scars and parastichies (Fig. 4C, D). The stem perimeter (*P*) systematically diminishes (Fig. 4F) and shows a breakpoint at 6.9 m height. Scar widths (*Sw*) show a slight reduction up to a breakpoint at 6.3 m height, after which their size begins to decrease considerably (Fig. 4G). Scar to stem ratio (*SSratio*) shows a breakpoint in between, at 6.7 m height (Fig. 4H). Internode length (*IL*) (Fig. 4J) shows a narrow section in the upper part of the bole. A phyllotaxis pattern transition from (8,5) to (8,13) parastichy pairs take place at approximately 9.0 m height (Table 3; Fig. 4D). In the adult specimen, there is no evident change in the divergence angle along height (Fig. 4I). Indeed, the linear model showed a slope close to zero (p-value 0.195).

The analysis of stem parameters for each of the main phyllotaxis sections (Table 4) shows that scar width (*Sw*), stem perimeter (*P*) and the Scar to Stem ratio (*SSratio*) are significantly larger in section (8,5). Conversely, internode length (*IL*) is significantly reduced from 2.7 cm in section (8,5) to 0.9 cm in section (8,13). Divergence angles did not differ significantly between the two phyllotaxis sections (Table 4; Fig. 4I).

### Divergence angle (*d*) analysed by grouping criteria

The Kruskal–Wallis test of the divergence angle of the five specimens did not show statistically significant differences between the classes of parastichy pairs (3,2), (3,5), (8,5), (8, 13) [H(4) = 4.7567, p-value = 0.3132]. Similarly, the divergence angle in the adult specimen did not show significant differences between the classes of parastichy pairs (8,5) and (8,13) [H(1) = 0.078952, p-value = 0.7787].

## Discussion

### Phyllotaxis of *J. chilensis*

Two phyllotactic parameters of the Chilean palm, divergence angle and parastichy series, were measured in young and adult specimens to determine phyllotactic transitions.

The average divergence angles among all *J. chilensis* specimens ranged between 128.8° and 143.2°, with a global mean of 136.9°. This value is close to the golden angle (≈ 137.5°) associated to most spiral phyllotaxis patterns [21]. Additionally, this average divergence angle is close to the range of other palm species. In *Cocos nucifera*, the divergence angle is between 136° and 137.9° [9]; in *Euterpe oleracea* it is between 126.9° and 135.8° [10], and in *Calamus longipinna* it is about 137.5° [54]. The section of the adult specimen of *J. chilensis* with the (8,5) parastichy pairs (below 9.0 m height) had a mean divergence angle of 137.0° (± 0.84 SD), which is in the range described by Mitchinson [14] for plants with contacts 5 and 8 (136.8° ± 1.8 SD); this finding is also consistent with results from other palm species [10, 24]. The mean divergence angle was very stable, without significant differences between different specimens or along the stem of the adult individual, with a trend to show a lower coefficient of variation in older specimens and/or sections (Table 3). Spiral phyllotaxis occurs in most plants [14, 16, 19], since divergence angles close to the golden angle (~ 137.5°) reduce leaf shading along the stem [5, 25] and maximizes the exposure of leaves to sunlight [24], thus optimizing light interception. This angle has been also defined as the optimal one to minimize the energy cost of phyllotactic transition [2]. Optimized distribution of leaf vascular connections along the stem has also been identified as an advantage of spiral phyllotaxis [55].

Phyllotaxis may vary over the lifespan of *J. chilensis* (Table 3), as it has been noted for other species [56]. Thus, the paired opposite arrangement of cotyledons in a seedling may be followed by changes to give a spiral, helical arrangement of progressively higher order [55], as seen, for example, in the sunflower (*Helianthus annuus*). The younger *J. chilensis* specimens, between 4 and 7 years old, had a (3,2) phyllotaxis, while an older specimen, 13 years old, had followed to a (3,5) parastichy pair pattern. In the adult specimen, an (8,5) phyllotaxis in the lower part of the stem, shifted to an (8,13) as the palm reached 9.0 m in height. This last region corresponded to a narrower section of the stem, which started its formation when the reproductive phase of the species began. All the parastichy pairs were consecutive numbers of the Fibonacci sequence, as observed in other species by several authors [14, 15, 17–19, 24]. For younger individuals, the results are similar to those obtained by Barabé et al*.* [10] for *E. oleracea,* with a conspicuous (2,3) parastichy pair at the shoot tip. Rees [8] indicates that a 12.5-year-old specimen of *Elaeis guineensis* (oil palm) (already in the reproductive phase) has a parastichy pair of (8,13), with certain variations along the stem. This is similar to the (8,13) parastichy pair determined for the upper part of the adult specimen of *J. chilensis*. The main difference between *E. guineensis* and *J. chilensis* is the age at which the species present the (8,13) parastichy pair. In the case of the Chilean palm, the narrower part of the stem begins its formation after the plant has reached 40–60 years old, which is the estimated age for the beginning of the reproductive phase [26].

Throughout its lifespan, the phyllotaxis pattern of the Chilean palm changes, parastichy pairs increasing in higher order numbers of the Fibonacci series, as expected according to Jean [15]. At some point, when the palm is between 7 and 13 years old, the phyllotaxis changes from (3,2) to (3,5) parastichy pair, while the palm still has a small stem that will grow in diameter over the next ten or more years. Bryntsev [52] has reported changes in phyllotaxis as the stem grows in many species, and Atela [5] proposed a model of plant pattern formation that physically includes the appearance of primordia and the expansion of meristems. Bryntsev [52] also explained how the phyllotactic pattern remains constant when the stem stops growing in diameter, i.e., when the meristem stops expanding (for more explanations see Figs. 5 and 8 and the explanation in Atela [5]).

Based on the literature and our results, we also argue that at some point, when the specimen reaches its maximum diameter and it begins to increase in height, the change in the parastichy pair, from (3,5) to (8,5) occurs and remains constant until the stem gets narrow. This particular transition was not fully captured by our data, as the (3,5) section remains “hidden” at the base of the adult specimen, in a small section of undetectable scars in old individuals, sometimes buried at the base of the specimens. Only a small fraction was observed at the base of the adult specimen. Sampling individuals aged 20 to 30 years might reveal this transition stage.

Phyllotaxis is a useful feature to differentiate between the juvenile vegetative phase and the reproductive adult phase [57]. Accordingly, we propose that *J. chilensis* is in the juvenile/establishment phase when it presents (2,3), (3,2) and (3,5) parastichy pairs. In this phase, the stem of the Chilean palm is still in the process of increasing in diameter, which occurs during approximately the first 20 years of its lifespan, before the increase in height begins [51]. According to Tomlinson [25], four structural features of palms begin to change in this phase: internode length is short, but successive internodes are progressively wider, the transition to the adult phase is marked by longer internodes; leaves are progressively larger and more elaborate (the transformation from simple to pinnate leaves); adventitious roots show progressive increases in numbers and diameter; and increases the number of vascular stem bundles. The next phase is marked by changes in phyllotaxis, with the change from (3,5) to (8,5) parastichy pair, i.e., when *J. chilensis* enters the adult vegetative phase. In this phase, the stem reaches its maximum diameter and starts growing in height. This increase in height is due to the production of a short stem segment with each new leaf [25]. The last phase is the adult reproductive phase, which occurs with the narrowing of the stem [58, 59] and, according to our observations, with the change in parastichy pairs from (8,5) to (8,13). In this adult phase, flower production begins and, consequently, the ability to bear fruit [25]. In the case of *J. chilensis*, pleonant flowering begins as a single axillary shoot of flowering branches (inflorescences), but it does not interrupt the vegetative extension of the species [25]. Parastichy pair transformations occur over the lifespan of the Chilean palm due to the appearance of primordia and the effect of the meristem expansion on continuous transitions [5, 60], and not due to changes in the divergence angle.

Analysis of stem parameters contrasting the two main stem sections with (8,5) and (8,13) phyllotaxis (Table 4) indicates that these sections are characterized by a significant reduction of the scar width *Sw*, a reduction in stem perimeter *P* and a relatively greater reduction in the scar-to-stem ratio *SSratio*. These changes may not necessarily be a causal effect related to the generation of phyllotaxis patterns, since they are also part of the vegetative growth pattern of palms, as described by Tomlinson [25]. However, the significant reduction in internode length *IL* with the phyllotaxis transition to the (8,13) pattern, may be related to a more compact shape of the meristem, where more primordia interact with each other, generating a more complex and of higher-order spiral phyllotaxis, in agreement with the widely accepted hypothesis of inhibitory fields [21, 55] explained by auxin derived cell expansion [56].

This study of the phyllotactic parameters of *J. chilensis* is a contribution towards the determination of its phyllotaxis and the characterization of morphological changes in its lifespan. Despite the small sampling size due to restrictions on the use of the scanning equipment, our results were consistent with previously reported findings in other palm tree species. Future research should include a larger number of samples and should involve different natural populations.

### The contribution of MRI and TLS to the study and the proposed methodologies

We proposed a novel use of non-destructive technologies for measuring the phyllotaxis in young and adult specimens of palm trees, based on MRI and TLS, which might contribute to the development of future studies in *J. chilensis* or other species.

Using MRI allowed us to identify the particularly close arrangement of leaf structures at the apex in a young specimen of *J. chilensis.* This was achieved by analyzing different axial images of the 3D data. The differences in water content within each leaf, and the presence of void sectors between consecutive and overlapping leaves, clearly marked the limits between structures. Additional MRI-based contrast mechanisms, such as differences in relaxation parameters (e.g., T1, T2), might also contributed to enhance structure visualization. Future work might quantify those relaxation parameters to further characterize this species. The samples were living, turgid and functioning plants and our scanning process guaranteed not to alter the shape and size of the structures. MRI is one of the most precise non-destructive tools for acquiring images and scanning internal structures, allowing the spiral arrangement of the outer visible leaves and the leaf primordia at the apical meristem to be measured with high detail. In this sense, the images obtained in our research demonstrated the suitability of MRI to detect tightly packed structures, such as unfolded leaves at the apex of palms species. MRI has already been used in phyllotaxis analyses, but looking inside the wood, such as the stems of cherry (*Prunus avium* L.) [61] or the branches and cones of *Pinus radiata* [29]. MRI allows showing a wide variety of physical, chemical, or physiological properties, which can be set up by modifying the acquisition parameters. In our study, we chose those parameters that enabled us to distinguish clearly anatomical structures. Other MRI sequences and parameters might be chosen to analyze chemical compositions, or even water diffusion, that might open a window to study more complex physiological processes. However, MRI exhibits major restrictions in terms of the size and handling of the samples. Standard clinical MRI scanners have bore diameters between 60 and 80 cm, and samples must be introduced lying down into the scanner, hindering the study of large specimens.

No references were found related to the use of TLS for phyllotaxis detection of standing adult palm specimens or for the quantification of the position and size of the scars. TLS allows the acquisition and analysis of detailed morphological features [62]. Nevertheless, most of the research performed with TLS and reported in the literature has focused on measuring the volume and branching of trees. In palm trees analyses, TLS has been used mainly for volume and biomass computations, although some studies have used it to detect changes in palm crown architecture and to detect fungal infections [63–65].

Our proposed methodology, i.e., scar recognition on a planar projection of the stem surface and further measurements of size and position, contributes to acquiring information of the previous evolution of an individual (as dendrochronology does in trees), based on a commonly dismissed morphological feature. The scar information we used in this study might also help understand the growth rate of the species and intra-annual growth cycles. Measurements of the scar width, required to determine the divergence angle, were accompanied by measurements of the internode length, and height and area of the scar. In data not shown, those variables presented periodic changes in size that might be associated to growth cycles. For instance, we observed periodic changes in scar size with cycles of 9 to 12 contiguous leaves.

Using TLS technology, as we propose, is an effective way to overcome the difficulties of analyzing standing individuals [25, 66]. This technology allows acquiring valuable information from the stem surface of palm trees, but it might be also used for other species. TLS stem surface scanning has been applied to study wood quality of other species [44, 67]. In our case, the use of TLS data and the proposed algorithm allowed us to identify precisely scars and their boundaries, despite of the relatively smooth surface of the *J. chilensis* stem. Therefore, the proposed methodology should work adequately for palms with larger and more protuberant scars, such as *Phoenix canariensis*, *Washingtonia robusta*, among others.

However, TLS presents some important restrictions that should be considered. It can only scan surfaces. Any internal structure of interest must be studied with tomographic scanners such as MRI or Computerized Tomography. In addition, any interfering object between the equipment and the specimen under analysis will preclude data acquisition, as it happens with branches and leaves blocking access to the stem surface at the top of the stem under the crown. Therefore, complementary technologies and methodologies should be considered.

## Conclusions

The Chilean palm tree *J. chilensis*, has features that make it special among all species of its family. The species shows a divergence angle throughout its lifespan that is close to the golden angle and remains stable at different developmental stages of the species. The identified parastichy pairs in our samples are consecutive pairs of the Fibonacci sequence, changing from (2,3) or (3,2), to (3,5) or (5,3), to (8,5), and to (8,13), as development progresses from the juvenile to the adult reproductive phase.

One of the main results of our study is the relationship between the lifespan phase of the palm and its phyllotaxis. This is relevant since no methods have been developed yet to determine the age of a palm tree [66]. Therefore, the relationship between developmental phases and the phyllotaxis that we described might be useful when studying the structural dynamics of natural populations of *J. chilensis* or other palm species.

In the context of uncertainty about climate change and potential threats to almost all species, a better understanding of the growth and developmental characteristics of any species is part of the general knowledge we require to model future populations under new environmental scenarios. In this sense, we hope we have contributed with new information about this endangered endemic species.

Finally, the two proposed technologies, MRI for seedlings and TLS for standing specimens, and the proposed analysis methodologies are well suited for non-destructive phyllotaxis research and analysis, and therefore, further use is encouraged in the search of other plant features.

## Data Availability

Data available on request from the authors.

## Abbreviations

MRI: Magnetic Resonance Imaging
TLS: Terrestrial Laser Scanning
RF: Radio-Frequency
LiDAR: Light detection and ranging
TR: Repetition time
DGA: General Water Office (Dirección General de Aguas)
LP: Lattice point
